# Individual Differences in Error‐Related Brain Activity and Post‐Error Slowing in Children

**DOI:** 10.1002/hbm.70581

**Published:** 2026-06-19

**Authors:** Gülce Akin, Sina A. Schwarze, Ulman Lindenberger, Silvia A. Bunge, Yana Fandakova

**Affiliations:** ^1^ Department of Psychology University of Trier Trier Germany; ^2^ Center for Lifespan Psychology, Max Planck Institute for Human Development Berlin Germany; ^3^ Max Planck UCL Centre for Computational Psychiatry and Ageing Research Berlin Germany; ^4^ Department of Psychology and Helen Wills Neuroscience Institute University of California at Berkeley Berkeley USA

**Keywords:** anterior insula, cognitive control, dACC, development, error monitoring, post‐error slowing, task switching

## Abstract

Errors play a crucial role in learning and goal‐directed behavior by triggering cognitive adjustments to optimize future task performance. One such adjustment is post‐error slowing (PES), the tendency to respond more slowly after an error. In adults, PES has been associated with regions implicated in error processing, including the anterior cingulate cortex (ACC) and anterior insula. The prolonged maturation of these regions is thought to contribute to less efficient error processing in children and PES compared to adults. Additionally, while some errors may be immediately corrected, resulting in isolated errors, others may require multiple correction attempts, resulting in consecutive errors. Compared to adults, children may need more attempts to correct their errors due to the ongoing neurodevelopment of error processing. We investigated age differences in error types and in PES between children (*N* = 159, 8–11 years) and adults (*N* = 40, 20–30 years) during task switching. We tested whether individual differences in error processing‐related activation contributed to PES within a subsample of children that performed the task during scanning (*N* = 72). Children made mostly consecutive errors, whereas adults made mostly isolated errors. PES magnitudes were larger in adults than in children. Children showed enhanced error‐related activity in dorsal ACC and the anterior insula. Enhanced error‐related activity in the insula was associated with better performance and reduced switch costs. These findings suggest that the neurodevelopment of error processing in late childhood contributes to the improved ability to adjust behavior following errors, and consequently to task‐switching performance.

## Key Points

- During task switching, children produced longer series of errors, whereas adults were more likely to correct their errors immediately after committing them. Unlike adults, children did not slow down after making an error to correct their performance.
- Among children, those who demonstrated greater post‐error slowing were more likely to show higher task‐switching performance, suggesting adaptive post‐error adjustments in children.
- During error processing, children showed increased activation in the dorsal anterior cingulate cortex and the anterior insula. Error‐related insular activation was associated with improved task performance, indicating that enhanced error processing contributes to adaptive performance adjustments during task switching.

## Graphical abstract

During task switching, children produced longer error series and showed less post‐error slowing than adults. During and after errors, children showed increased activation in cingulate, insular, and prefrontal regions. Children's post‐error slowing and error‐related insular activation were associated with improved performance, indicating that error processing contributes to adaptive performance adjustments.

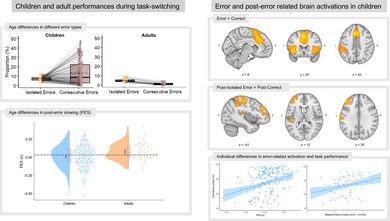

## 1 Introduction

Cognitive control refers to a set of processes that enable humans to regulate their behaviors and thoughts toward specific goals, allowing them to adapt to changing environments (Bunge and Crone 2009; Diamond 2006; Inzlicht et al. 2015; Miller and Cohen 2001). One key aspect of cognitive control is cognitive flexibility, or the ability to quickly adjust to new task rules or demands including switching between them (Diamond 2013; Monsell 2003). Shifting between different tasks or contexts is typically associated with switch costs, which are operationalized as slower response times and increased error rates when a task rule changes from one trial to the next, as opposed to when the same rule is repeated across trials (Monsell 2003). Switch costs are thought to reflect the inhibition of the previous task set and the retrieval of the currently relevant one (Meiran 1996; Mayr and Keele 2000; Monsell 2003).

Cognitive control processes continue to develop throughout childhood and adolescence (Bunge et al. 2002; Luna et al. 2004; Tervo‐Clemmens et al. 2023) including the ability to flexibly switch between tasks (Ger and Roebers 2023; Gupta et al. 2009; Kray et al. 2004; Schwarze et al. 2024). Compared to adults, children tend to show longer response times and lower accuracy when switching between tasks compared to when tasks repeat, resulting in higher switch costs. Switch costs decline with age and reach adult levels by around 9–11 years of age (Cepeda et al. 2001; Chevalier et al. 2015; Crone et al. 2006; Schwarze et al. 2023). Appropriate task‐switching is supported by the ability to adjust performance after an error; as discussed below, this aspect of cognitive control, too, shows prolonged maturation.

The ability to detect and avoid errors is crucial for improving performance, especially in tasks that are prone to errors due to high demands on cognitive control (Holroyd and Coles 2002; Ullsperger et al. 2014). One commonly observed behavioral adjustment is post‐error slowing (PES), where individuals tend to slow down (i.e., show increased response times) after committing an error (Notebaert et al. 2009; Rabbitt 1966). PES has often been interpreted as an adaptive process, as it is often positively associated with accuracy on trials immediately following an error (Gehring and Fencsik 2001; Ridderinkhof et al. 2004). Larger PES is thought to reflect response caution following an error, allowing individuals to collect more task‐relevant information before the next decision or action (Dutilh et al. 2012; Wessel 2018), especially in dynamic environments where demands change frequently (Danielmeier and Ullsperger 2011; Dutilh et al. 2012; Marco‐Pallarés et al. 2008; Rabbitt 1966). These adaptive processes lead to more deliberate and controlled responding that reduces errors.

However, PES is not always associated with increased accuracy; therefore, it has alternatively been described as a maladaptive process that leads to performance disruptions (Buzzell et al. 2017; Notebaert et al. 2009; Purcell and Kiani 2016). By this account, PES causes interference through the distraction of attention, diverting cognitive resources away from the task at hand. For example, errors could serve as infrequent and surprising events that can trigger negative emotional reactions such as frustration, anger, or embarrassment (Dutilh et al. 2012; Pfister and Foerster 2022). These reactions are thought to impair task performance through their negative effects on attention, leading to decreased accuracy (Buzzell et al. 2017; Notebaert et al. 2009).

Taken together, the adaptiveness of PES remains a topic of ongoing debate (Gjorgieva and Egner 2022; Wessel 2018). Different adaptive and maladaptive processes associated with PES have been reconciled in a framework (Wessel 2018) that distinguishes between strategic PES and orienting‐related PES. Strategic PES is defined as a slow, deliberate adjustment after an error that serves to improve future performance. In contrast, orienting‐related PES is thought to occur when there is insufficient time to fully process the error before a new task begins, leading to delays without necessarily improving accuracy. This account thus suggests that the timing between subsequent trials (i.e., inter‐trial intervals; ITIs) may be critical for promoting adaptive PES processes.

If PES is an adaptive mechanism, it might help correct errors effectively on the next try. However, while some errors can be corrected immediately, it may take multiple attempts to correct an error when PES‐related processes cannot be engaged efficiently (Hajcak and Simons 2008). To date, only a few studies in adults have distinguished between different types of errors (e.g., Hajcak and Simons 2008; Hester et al. 2007). Of note, Hajcak and Simons (2008) reported positive associations between PES and both double errors (i.e., PES for an error following an error) and single errors (i.e., PES for a correct trial following an error). Notably, the magnitude of the association was larger for single errors, suggesting that double errors may be related to failures in implementing post‐error behavioral adjustments.

The ability to learn from mistakes, including slowing down after errors, emerges early in life and undergoes significant changes during childhood and adolescence (Brewer and Smith 1989; Smulders et al. 2016; Tamnes et al. 2013). Even young children between 3 and 6 years slow down following errors, although this slowing is not always associated with improved performance (Ger and Roebers 2023; Jones et al. 2003). Most developmental studies have reported age‐related decreases in PES magnitudes from childhood through adolescence, for example, across ages 5–12 (Fairweather 1978; see also Dubravac et al. 2022) and ages 7–16 years (Schachar et al. 2004). Further studies have shown age‐related decreases in PES from childhood to adolescence and adolescence to adulthood (e.g., Brewer and Smith 1989 [5–11 years]; Carrasco et al. 2013 [10–17 years]; Smulders et al. 2016 [5–25 years]). These findings suggest a highly protracted PES development across childhood, adolescence, and young adulthood, potentially reflecting more refined and efficient adjustments in response times (Brewer and Smith 1989; Thaqi and Roebers 2020; Schachar et al. 2004).

However, some studies have also reported non‐linear patterns of PES across development, or no age‐related difference. For instance, although Gupta et al. (2009) found an overall decrease in PES from ages 7–10 years in a task‐switching paradigm, they observed an initial increase from 6 to 7 years followed by a slight decline between 7 and 8 years, and a more pronounced reduction from 9 to 10 years (see also de Mooij et al. 2022). These findings suggest that developmental changes in PES may follow complex, non‐monotonic maturation processes rather than simple linear improvements with age. Finally, some studies also reported larger PES in adults than in children (Friedman et al. 2009 [approximately 10 and 24 years]; Hogan et al. 2005 [12–18 and 18–22 years]) or found comparable PES across childhood, adolescence, and adulthood (Davies et al. 2004 [7–18 and 19–25 years]; Ladouceur et al. 2004 [9–14 and 14–17 years]; Wiersema et al. 2007 [7–8, 14–17 and 23–24 years]).

This mixed pattern of results may stem from differences in the precise age ranges and sample sizes across studies; additionally, they may be related to experimental variations such as differences in task demands, stimulus duration, and inter‐trial intervals. Although methodological differences make it challenging to draw overarching conclusions about the development of PES, a common pattern emerges such that the majority of studies reporting age‐related decreases in PES tend to have either longer stimulus durations (in line with Wessel 2018), resulting in extended response times or designs with self‐paced responses (de Mooij et al. 2022; Dubravac et al. 2022; Ger and Roebers 2023; Gupta et al. 2009; Jones et al. 2003; Schachar et al. 2004). In contrast, studies reporting age‐related increases or no differences in PES typically impose more restricted stimulus presentation times (Davies et al. 2004; Friedman et al. 2009; Hogan et al. 2005; Wiersema et al. 2007).

From a developmental perspective, these divergent findings may reflect different facets of the ongoing maturation of cognitive control. Under strict time limitations, children's limited control capacity may prevent them from fully processing errors and making efficient behavioral adjustments; therefore, they may struggle to engage in strategic PES, resulting in not slowing down after errors. In contrast, when sufficient time is available, children's still‐developing cognitive control might support error processing and engagement in strategic PES, albeit not at the level of adults, resulting in longer PES in comparison to adults.

The protracted development of PES may reflect the ongoing maturation of the brain areas involved in error processing and PES (Fitzgerald et al. 2010; Santesso and Segalowitz 2008; Schachar et al. 2004; Tamnes et al. 2013). Previous fMRI research in adults has linked post‐error behavioral adjustments to activation in the anterior cingulate cortex (ACC; Debener et al. 2005; Garavan 2002; Kerns et al. 2004), lateral prefrontal cortex (PFC; Garavan 2002; Hester et al. 2007; King et al. 2010; Li et al. 2008; Zhang et al. 2017), posterior medial frontal cortex (pMFC; Hester et al. 2007), and anterior insula (Zhang et al. 2017). The ACC, with activation extending into adjacent regions along the medial wall, is consistently activated during interference and error processing (Fitzgerald et al. 2010; Botvinick et al. 2001; Carter et al. 1998; Ridderinkhof et al. 2004). Beyond error detection, the ACC is thought to play a pivotal role in triggering reactive control adjustments following errors, helping to improve subsequent task performance by recruiting additional prefrontal regions (Kerns et al. 2004; Garavan 2002; Sheth et al. 2012). Beyond the ACC's general role in error detection and performance monitoring, higher error‐related ACC activity has been directly linked to greater PES in adults (Kerns et al. 2004; King et al. 2010; Klein et al. 2007). The anterior insula also plays a role in error processing. It is particularly important for detecting salient events, such as errors, and helps maintain task‐relevant information, and coordinate cognitive control with the ACC, PFC, and parietal regions (Dosenbach et al. 2007; Klein et al. 2007; Menon and Uddin 2010; Ullsperger et al. 2010). Importantly, increased activity in the anterior insula (Zhang et al. 2017), PFC (Li et al. 2008), and pMFC (Cavanagh et al. 2009; Debener et al. 2005) has been positively associated with PES in adults.

Taken together, the ACC and anterior insula have been consistently implicated in processing errors and in guiding the adjustments that follow, supporting performance correction and optimization (Menon and Uddin 2010; Ridderinkhof et al. 2004; Ullsperger et al. 2010). However, the extent to which these regions contribute to error‐related adjustments in childhood is still not well understood. This question is particularly pertinent since the ACC and anterior insula become increasingly specialized and functionally integrated from childhood through adolescence, contributing to cognitive development (Barber et al. 2013; Dennis et al. 2013; Fandakova et al. 2017; Hasan et al. 2009; Kelly et al. 2009). For instance, children who exhibited greater anterior insula activation when erroneously remembering information during memory retrieval also showed longitudinal increases in prefrontal activation and in memory performance over the course of approximately one and a half years (Fandakova et al. 2018).

Likewise, the ACC, particularly the dorsal region, shows increasing engagement in service of cognitive control and performance monitoring from childhood to adulthood (Crone 2014; Peters et al. 2014; Rubia et al. 2006). Furthermore, electrophysiological markers like the error‐related negativity (ERN), a negative deflection on the scalp following an error, which originates in the ACC (Davies et al. 2004), become more pronounced with age across childhood and adolescence (Carrasco et al. 2013; Davies et al. 2004; Hanna et al. 2012; Ladouceur et al. 2004; Santesso and Segalowitz 2008; Wiersema et al. 2007). Together, these findings indicate a protracted maturation of neural systems supporting error processing (Santesso et al. 2006; Kang et al. 2021) that may contribute to the emergence and refinement of adaptive post‐error adjustments like PES during development.

In the present study, we aimed to investigate whether error‐related brain activity in children is associated with post‐error slowing and task‐switching performance. As a first step, we examined PES in relation to overall task performance and different types of errors in children aged 8–11 years (*N* = 159) and adults (*N* = 40) who performed a task‐switching paradigm either behaviorally or during fMRI data collection. Given the mixed findings in previous developmental research, we further examined age differences in PES during task switching and whether it contributed to better task performance, thus testing its adaptive nature. It is well‐established that children make more errors and exhibit longer response times compared to adults (Davies et al. 2004; Davidson et al. 2006; Luna et al. 2004). However, to the best of our knowledge, no study to date has examined if adults and children differ in the types of errors they commit, such as consecutive errors that are corrected after multiple attempts, or isolated errors that are corrected immediately on the following trial. To close this knowledge gap, we aimed to investigate how isolated and consecutive errors differ between adults and children.

The dataset used in the present study was originally collected as part of a larger project investigating task‐switching training in children (Schwarze et al. 2023, 2025, 2026). These studies provide a comprehensive account of the development of task switching and its modulation through training. While these previous analyses focused on task‐switching costs and did not examine the effects of errors, here we set out to examine post‐error processes in an exploratory manner, including how children commit errors and how they recover from them.

We then tested whether brain activation during errors and on trials following an error was associated with PES and task‐switching performance in a subsample of children who underwent neuroimaging during the task‐switching paradigm. Based on the previously documented involvement of ACC and anterior insula in error processing both in children and adults (Buzzell et al. 2017; Denervaud et al. 2024; Tamnes et al. 2013), we predicted higher activation in these regions during errors in comparison to correct responses in children. Given the ongoing development of performance monitoring processes in childhood linked to ongoing ACC and insular maturation (Tamnes et al. 2013), we explored whether children who exhibited more enhanced error‐related activity would also demonstrate greater PES and better task‐switching performance.

## 2 Methods

### 2.1 Participants

#### 2.1.1 Behavioral Analyses

A total of 185 children (8–11 years, *M* = 9.9 years, SD = 0.7 years; 89 girls, 96 boys) and 53 adults (20–30 years, *M* = 24.6, SD = 2.6; 28 women, 25 men) who participated in a task‐switching training study were included in behavioral analyses (see Schwarze et al. 2023, for details of the study). For each participant, responses shorter than 250 ms (i.e., to minimize accidental responses, Ger and Roebers 2023) or longer than 3000 ms were excluded from the analyses (resulting in a total of 1% of excluded data). Participants with less than 40% overall accuracy during task switching were also excluded (*N* = 9 children). As we were interested in examining PES, participants were excluded if they had fewer than 5 isolated errors, defined as errors following and followed by correct responses (*N* = 13 adults, *N* = 17 children). From the remaining data, we tested for outliers in average response times (RTs) in each condition of interest. In each age group, there were no outliers identified at *p* < 0.001, two‐tailed (Tabachnik and Fidell 2013). The final sample thus included 159 children (*M* = 10 years, SD = 0.7 years; 76 girls, 83 boys) and 40 adults (*M* = 24.6 years, SD = 2.7 years; 20 women, 20 men). The study received approval from the ethics committee of the Freie Universität Berlin and was conducted in accordance with the Declaration of Helsinki.

#### 2.1.2 fMRI Analyses

From the initial dataset of 185 children, 64 children completed the task only behaviorally in the mock scanner. Data from 6 children were excluded due to technical reasons and 21 children were excluded due to excessive in‐scanner head motion, defined as more than 50% of fMRI volumes with framewise displacement exceeding 0.4 mm (Power et al. 2012; see Schwarze et al. 2023 for details). From the remaining participants, we excluded participants with less than 40% accuracy and less than 5 trials for relevant conditions of interest (see fMRI analyses: general linear models (GLM) 1 and 2) per run. For the analysis of error‐related brain activity, this criterion resulted in the exclusion of 18 children and a final sample of 72 children (*M* = 10.05 years, SD = 0.7 years; 40 girls, 32 boys). For the analysis of post‐error‐related brain activity, 24 children were excluded, resulting in a final sample of 48 children for analysis (*M* = 10.06 years, SD = 0.8 years; 30 girls, 18 boys).

### 2.2 Experimental Design

**FIGURE 1:**
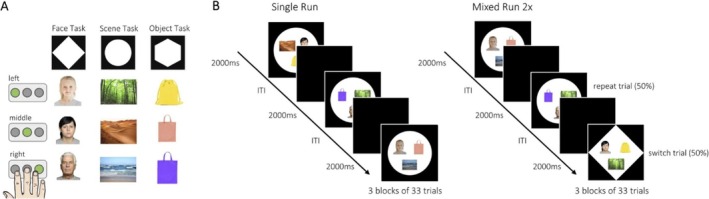
Experimental design. Adapted from Schwarze et al. (2023), Figure 1, under CC.BY 4.0.

Participants completed a task‐switching paradigm that consisted of three categorization tasks. On each trial, the relevant task was cued by one of three geometrical shapes in the background of the screen (see Figure 1A). Each trial displayed a face, a scene, and an object overlaid on the task cue. In the face task (cued by a diamond), participants had to decide if the face depicted an image of a child, a young adult, or an older adult. In the scene task (cued by a circle), participants had to decide if the scene depicted a forest, a desert, or a sea. Finally, in the object task (cued by a hexagon), participants classified a bag according to its color (i.e., yellow, red, or purple). The images appeared simultaneously with the cue for 2000 ms and were followed by a jittered fixation cross (1000–6000 ms). The spatial arrangement of the images within the task cue frame varied randomly on each trial and was independent of the categorization rule (see Schwarze et al. 2023, for further details).

During a pretest session, participants were familiarized with the task through instructions and practice on both single runs that included feedback and mixed runs without feedback. On the day of testing, prior to the main task, they completed additional practice on single runs with feedback, followed by a mixed run without feedback. Participants did not receive any feedback during the main task.

Participants first completed three runs of 99 trials each. The first run was a single run, in which the three tasks (face, scene, and object categorization) were presented in separate blocks of 33 trials, and no switching between was required. The second and third runs were mixed‐task runs, with tasks intermixed such that the task rule repeated on ca. 50% of the trials and switched on the remaining ca. 50% of the trials (see Figure 1B). Only data from the mixed‐task runs were analyzed here because these runs required participants to alternate between the three tasks, allowing for the investigation of error processing during task switching. The first trial of each block was excluded from the analyses, as it could not be classified as either switch or repeat trials.

### 2.3 Behavioral Data Analyses

First, we investigated age differences between adults and children in terms of accuracy and RTs. Overall accuracy was calculated as the proportion of correct responses relative to the total number of responses given (correct and incorrect) across repeat and switch trials. To compare accuracy between groups, we conducted an independent‐samples *t*‐test. To examine whether children and adults differed in RTs on correct versus incorrect trials, we conducted a mixed‐design analysis of variance (ANOVA), with age group (children vs. adults) as a between‐subjects factor and accuracy (correct vs. incorrect) as a within‐subjects factor.

Second, we investigated age‐related differences in cognitive flexibility by examining switch costs in both RTs and accuracy. Switch costs in RTs were computed as the difference between switch and repeat trials, considering only correct responses (Wylie and Allport 2000). Switch costs in accuracy were calculated as the difference in proportional accuracy between repeat and switch trials. We performed two independent‐samples *t*‐tests to compare switch costs between adults and children for each measure. It should be noted that Schwarze et al. (2023) also reported analyses pertaining to switch costs. However, the present study and Schwarze et al. (2023) were based on only partially overlapping samples, as Schwarze et al. (2023) restricted analyses to participants with fMRI data. For this reason, we also reported switch‐cost analyses in the present study.

To explore potential speed–accuracy trade‐offs, we computed linear integrated speed–accuracy scores (LISAS) for each participant and each of the switch and repeat conditions. LISAS combine mean RTs and error rates into a single metric while preserving their separate contributions (Vandierendonck 2017). LISAS were calculated as participant's mean RT for correct responses in the corresponding condition (*j*: switch or repeat) plus the product of the participant's error rate (PE) in that condition and the ratio of the participant's standard deviation of correct RTs across conditions to the standard deviation of error rates across conditions: $\text{LISAS}=RT_{j}+\frac{SD_{\mathrm{RT}}}{SD_{\mathrm{PE}}}\times PE_{j\;}$. To examine age differences in LISAS, we conducted a mixed‐design ANOVA with the between‐person factor age group (adults vs. children) and the within‐person factor condition (repeat vs. switch).

Next, to assess how error processing differed between adults and children, we examined various types of error rates, including: (1) isolated errors, defined as errors that always follow correct responses and are immediately corrected, and (2) consecutive errors, defined as errors that are not corrected on the first attempt and require multiple attempts to correct. To do so, we categorized individual trials based on response sequences of interest: we categorized each element in a sequence of correct, incorrect, and then correct responses (i.e., 1 0 1) as follows: the initial correct response preceding the error was defined as the “pre‐error correct response” the incorrect response represents the “isolated error” and the last correct response following the error represents the “post‐error correct response”. In a sequence of multiple errors where the initial error was not corrected, but was instead followed by additional errors (i.e., 0 0 0), each error in the sequence was labeled as a “consecutive error”. Errors that did not fit the categories of isolated or consecutive errors (e.g., errors preceding or following a missing value) were not further considered in the present analysis (3.7% of all trials across participants). For each error type, rates were calculated as the number of responses of the corresponding type divided by the total number of all responded trials. Non‐response trials (12.8% of all trials across participants) were not included in the calculations. To examine age group differences in the rate of error types, we conducted a mixed‐design ANOVA with age group (children vs. adults) as a between‐subjects factor and error type (isolated errors vs. consecutive errors) as a within‐subjects factor.

Finally, we examined age differences in PES. In the literature, the magnitude of PES is commonly computed as the mean RT of correct trials following an error minus the mean RT of correct trials following a correct response (Overbye et al. 2019; Pfister and Foerster 2022; Smith et al. 2020). However, this calculation does not account for accuracy on the pre‐error trial. To control for possible effects of pre‐error speeding (see Pfister and Foerster 2022), we calculated PES as the difference between the mean RT of correct trials following errors in correct‐error‐correct sequences and the mean RTs of all correct trials following correct responses. More specifically, a correct‐error‐correct sequence consisted of three consecutive trials in which the participant first responded correctly, then made an error, and then responded correctly again. By restricting the analysis to sequences in which the post‐error trial was answered correctly, this approach specifically captured slowing down that co‐occurred with successful adjustment following errors, that is, trials on which the participant not only slowed down after making an error but also responded accurately on the immediately following trial. As a result, we conceptualized PES as an adaptive mechanism following isolated errors, reflecting a form of cognitive control that supports accurate performance after mistakes.

We then performed one‐sample *t*‐tests to determine whether the PES magnitudes for adults and children each differed significantly from zero. Subsequently, we compared PES between adults and children using an independent‐samples *t*‐test. To examine the relationship between PES and overall accuracy and switch costs, we computed Pearson correlations separately for children and adults. In a final set of control analyses, we performed a mixed‐design ANOVA to explore how PES is affected by inter‐trial intervals (ITIs, 1, 2, 4, and 6 s) as a within‐subjects factor and whether this effect varied by age group (children vs. adults) as a between‐subjects factor.

As an exploratory analysis, we tested more directly whether slowing was associated with subsequent accuracy in children. To this end, we conducted a generalized linear mixed‐effects model at the trial level, with accuracy on the current trial predicted by the interaction between current response time and previous trial accuracy.

All analyses were performed using R 4.5.1 (R Core Team 2025) with the packages cocor (Diedenhofen and Musch 2015), effectsize (Ben‐Shachar 2020), ez (Lawrence 2016), rstatix (Kassambara 2023), psych (Revelle 2025), lme4 (Bates et al. 2015). The significance level was set at *p* < 0.05 for all analyses. Corrections for multiple comparisons were applied using a Bonferroni correction.

### 2.4 fMRI Acquisition and Preprocessing

The present analysis is based on data reported by Schwarze et al. (2023); please see the original paper for details regarding image acquisition and preprocessing of the fMRI data. Briefly, anatomical and functional MRI data were acquired using a 3‐Tesla Siemens Tim Trio MRI scanner with a 32‐channel head coil. High‐resolution T1‐weighted structural images (220 slices, 1 mm isotropic voxels, TR = 4500 ms, TE 2.35 ms) were collected along with 230 whole‐brain echo‐planar images per run (36 interleaved slices, TR 2000 ms, TE 30 ms, 3 × 3 × 3 mm). Participant motion was monitored throughout the task using Framewise Integrated Real‐time MRI Monitoring (FIRMM; Dosenbach et al. 2007).

Preprocessing was conducted using fMRIprep (Version 20.2.0; Esteban et al. 2019). BOLD images were co‐registered to individual anatomical templates using FreeSurfer (Greve and Fischl 2009), slice‐time corrected using AFNI (Cox and Hyde 1997), and realigned using FSL 5.0.9 (Jenkinson et al. 2002). Images were resampled into MNI152NLin6Asym standard space (voxel size 2 mm isotropic). Finally, they were spatially smoothed using an 8 mm FWHM isotropic Gaussian kernel in SPM12 (Functional Imaging Laboratory, UCL, UK). The first five volumes of each run were discarded to allow for stabilization of the magnetic field.

### 2.5 fMRI Data Analyses

GLM 1. First, to investigate the neural correlates of error processing, we estimated GLMs at the single‐subject level using SPM12 (http://www.fil.ion.ucl.ac.uk/spm) including three regressors: correct trials (all correct responses), error trials (all incorrect responses), and a regressor of no interest including no‐response trials. Each stimulus was modeled as an event with a duration equal to the corresponding RT for each trial. Data were high‐pass filtered at 128 s. At the group level, one‐sample *t*‐tests were performed to assess the overall effects of the contrasts of error > correct and correct > error across all children included in the analyses (*N* = 72). Whole‐brain maps for each contrast were thresholded at *p* < 0.001 at the voxel level and corrected for multiple comparisons using cluster‐level false discovery rate (FDR) correction at *p* < 0.05.

GLM 2. Second, to investigate the neural correlates of post‐error related processing, we estimated GLMs including five conditions: correct trials, correct trials following correct trials (post‐correct), isolated error trials, correct trials following isolated errors (post‐isolated error), and a regressor of no interest including no‐response trials. At the group level, one‐sample *t*‐tests were performed to assess the average effects of the contrasts of post‐isolated error > post‐correct and post‐correct > post‐isolated error across all children included in the analyses (*N* = 48). Preprocessing parameters, GLM specifications, and statistical thresholding were the same as the criteria described above.

### 2.6 Region of Interest (ROI) Definition and Analyses

We performed region of interest (ROI) analyses to define brain regions that were significantly activated during error responses relative to correct responses and during post‐isolated error relative to post‐correct conditions. The ROI analyses allowed us to examine whether error‐related activity was associated with behavioral performance. For all ROIs, 6 mm spheres were constructed around the group‐level activation peaks of the regions that emerged in the whole‐brain comparison for error > correct or post‐isolated error > post‐correct (see Tables 1 and 2 for details). Spheres generation and beta parameter extraction were performed using MarsBaR (Brett et al. 2002). Extracted beta parameters were screened for outliers across participants at *p* < 0.001, two‐tailed, resulting in the removal of 0.6% of data for the error‐related analysis and 1.04% of data for the post‐error analysis. We combined the left and right anterior insula by averaging their activation values to reduce the number of comparisons, as both regions exhibited similar error‐related brain activity (error > correct, see Table 1).

**TABLE 1:** Results of whole‐brain fMRI analyses comparing errors and correct responses in children.

Contrast	Region	Cluster size	*Z* max	MNI coordinates (mm)		
				*x*	*y*	*z*
Error > correct	L dorsal ACC	**4548**	**6.84**	−**4**	**22**	**44**
			6.21	−14	4	68
			6.15	4	20	46
	L anterior insula/frontal operculum	**1839**	**6.20**	−**36**	**22**	−**4**
			6.01	−46	14	2
			5.56	−30	26	4
	R anterior insula/frontal operculum	**1183**	**5.61**	**34**	**22**	−**6**
			5.26	48	14	6
			4.83	34	22	6
	L supramarginal gyrus	**1392**	**4.62**	−**50**	−**34**	**40**
			4.32	−54	−32	48
			4.02	−64	−46	36
Correct > error	R Putamen	**2275**	**5.24**	**30**	−**12**	**0**
			5.19	8	22	6
			5.10	18	10	−8
	L Putamen	**1181**	**5.10**	−**28**	−**6**	−**10**
			5.10	−28	−10	0
			4.84	−20	10	−8
	L superior temporal gyrus	**654**	**4.44**	−**64**	−**26**	**6**
			4.10	−38	−38	2
			4.05	−62	−36	12
	L inferior occipital gyrus and pole	**1012**	**4.16**	−**22**	−**94**	−**2**
			3.91	−14	−90	−16
			3.91	−10	−100	−2

*Note:* Only cluster‐level FDR corrected regions included (*p* < *0.001, cluster‐level FDR corrected* (*p* < *0.05*)). Cluster maxima are indicated in bold, while local maxima are shown in regular font.

Abbreviations: L, left; MNI, Montreal Neurological Institute atlas; R, right.

**TABLE 2:** Results of whole‐brain fMRI analyses comparing correct responses following isolated errors (post‐isolated error) and correct responses following correct responses (post‐correct) in children.

Contrast	Region	Cluster size	*Z* max	MNI coordinates (mm)		
				*x*	*y*	*z*
Post‐isolated error > post‐correct	L postcentral gyrus	**2199**	**5.11**	−**56**	−**22**	**50**
			4.79	−46	−18	54
			4.61	−46	−26	14
	R cerebellum	**369**	**4.91**	**20**	−**52**	−**24**
	L middle frontal gyrus/frontal pole	**854**	**4.58**	**−40**	**38**	**30**
			4.07	−38	40	12
			3.96	−44	50	10
Post‐correct > post‐isolated error	L inferior occipital gyrus	**9866**	**4.81**	**−46**	−**78**	−**10**
			4.80	36	−70	−12
			4.72	−8	−84	−18

*Note:* Only cluster‐level FDR corrected regions included (*p* < 0.001, cluster‐level FDR corrected (*p* < 0.05)). Cluster maxima are indicated in bold, while local maxima are shown in regular font.

Abbreviations: L, left; MNI, Montreal Neurological Institute atlas; R, right.

We then computed Pearson correlations to examine the relationship between behavioral performance and error‐related and post‐error brain activation. To account for multiple comparisons, *p* values were adjusted using Bonferroni correction across all performed correlations. The significance was set at *p* < 0.05 for all correlational analyses.

For clarity, we note that throughout this article, “post‐error” and “post‐isolated error” are used interchangeably. Although we use the more specific term “post‐isolated error” when describing our analyses (which examined processing following isolated errors specifically), we use the more general term “post‐error” when discussing behavioral adjustments after errors more broadly.

## 3 Results

### 3.1 Age Differences in Overall Task Performance

**FIGURE 2:**
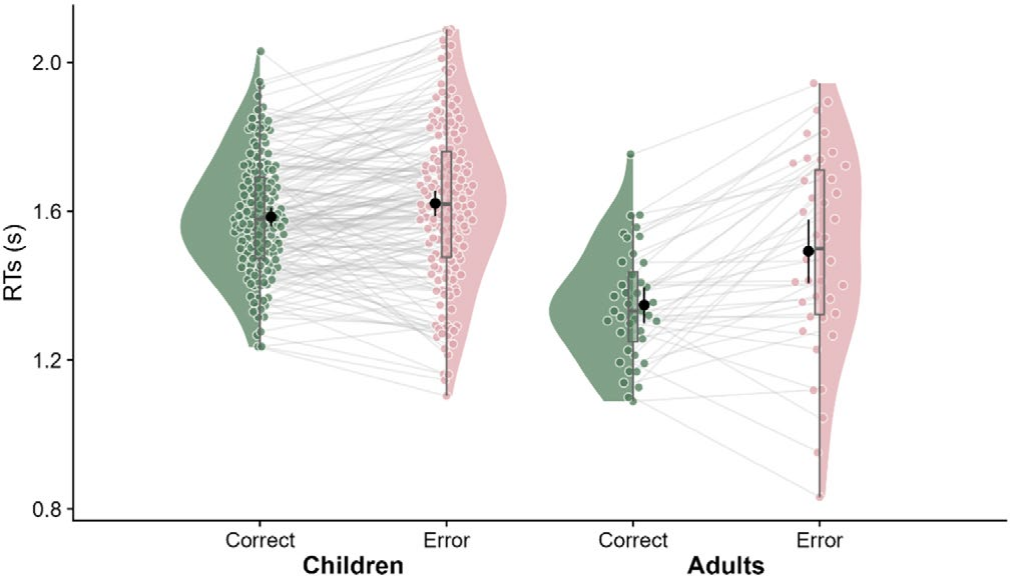
RTs for correct and error trials in children and adults. Each dot represents an individual participant connected by gray lines across conditions. Boxes show the interquartile range (IQR) with whiskers extending to 1.5 × IQR; horizontal lines within boxes indicate medians. Black dots represent means with 95% confidence intervals. Violin plots display response distributions.

First, we examined age differences in overall task accuracy collapsing across conditions of the task‐switching paradigm. An independent sample *t*‐test comparing overall accuracy between adults and children revealed that adults (*M* = 93%, SD = 3.45) performed significantly better than children (*M* = 74%, SD = 15.24), *t*(196) = 14.42, *p* < 0.001, *d* = 2.06. Next, we examined age differences in RTs for errors and correct responses collapsing across conditions of the task‐switching paradigm. A mixed‐design ANOVA with age group (children vs. adults) and response type (error vs. correct) revealed a significant main effect of age, *F*(1,197) = 33.28, *p* < 0.001, *η* ^2^ *p* = 0.145, indicating that adults were overall faster than children. Additionally, there was a significant main effect of response type, *F*(1,197) = 41.05, *p* < 0.001, *η* ^2^ *p* = 0.172, indicating that correct responses were overall faster than error responses. Finally, the interaction between age and response type was significant, *F*(1,197) = 14.69, *p* < 0.001, *η* ^2^ *p* = 0.069, indicating larger differences in RTs for adults in comparison to children (see Figure 2). In children, errors were significantly slower than correct responses, *t*(158) = 3.11, *p* = 0.002, *d* = 0.49. Adults also showed slower RTs for errors compared to correct responses, *t*(39) = 4.54, *p* < 0.001, *d* = 1.45. Taken together, both age groups demonstrated slower responses for errors, but the effect was relatively more pronounced in adults than in children.

### 3.2 Age Differences in Switch Costs

Next, we examined age differences in switch costs in accuracy and RTs. An independent sample *t*‐test comparing accuracy switch costs between children (*M* = 14.59, SD = 9.50) and adults (*M* = 5.39, SD = 3.36) revealed a significant difference between age groups, *t*(177) = 9.97, *p* < 0.001, *d* = 1.50, indicating that children showed greater accuracy switch costs than adults. For RT switch costs, an independent sample *t*‐test comparing children (*M* = 0.35, SD = 0.13) and adults (*M* = 0.39, SD = 0.08) revealed a significant difference between age groups, *t*(102) = 2.39, *p* = 0.01, *d* = 0.47, such that adults showed higher RT switch costs than children. To examine the extent to which these results may be related to potential speed–accuracy trade‐offs, we computed LISAS scores, which combine RTs and accuracy in a single metric. A mixed‐design ANOVA with age group (adults vs. children) and condition (repeat vs. switch) revealed a significant main effect of condition, *F*(1,197) = 960.17, *p* < 0.001, *η* ^2^ *p* = 0.830, indicating that LISAS scores were overall higher for the switch condition (*M* = 3.39, SD = 8.29) in comparison to the repeat condition (*M* = 2.70, SD = 8.36). However, the main effect of age, *F*(1,197) = 0.23, *p* = 0.63, *η* ^2^ *p* = 0.001 and the interaction between age and condition were not significant, *F*(1,197) = 2.40, *p* = 0.12, *η* ^2^ *p* = 012. Together, these combined scores suggest that children and adults showed comparable switch costs. However, children showed larger switch costs in accuracy, suggesting less effective switching. Therefore, we subsequently focused on switch costs in accuracy in children.

### 3.3 Age Differences in Patterns of Error Types

**FIGURE 3:**
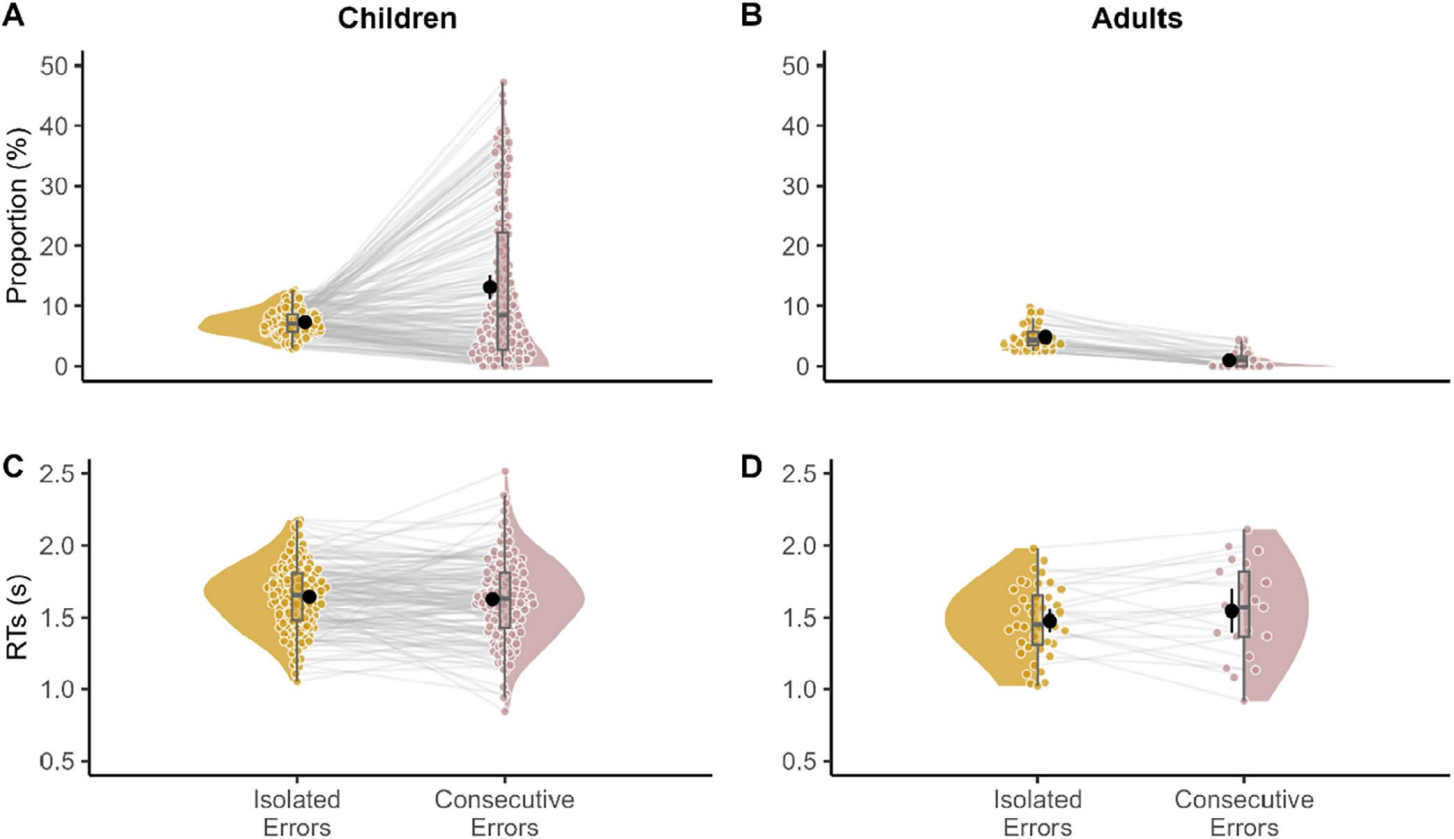
Percentage and RTs of isolated errors and consecutive errors in children and adults. Isolated and consecutive errors in children (A) and adults (B). RTs for isolated versus consecutive errors in children (C) and adults (D). Each dot represents an individual participant connected by gray lines across conditions. Boxes show the interquartile range (IQR) with whiskers extending to 1.5 × IQR; horizontal lines within boxes indicate medians. Black dots represent means with 95% confidence intervals. Violin plots display response distributions. Note that consecutive and isolated errors do not encompass all errors; those that did not fall into these categories were excluded from the analyses.

Next, we examined the percentage of different types of errors in children and adults. The results of mixed‐design ANOVA with age group (children vs. adults) and error type (isolated vs. consecutive) revealed a significant main effect of age group, *F*(1,197) = 46.53, *p* < 0.001, *η* ^2^ *p* = 0.191, consistent with the analyses above demonstrating overall lower accuracy in children compared to adults. The main effect of error type was not significant, *F*(1,197) = 1.14, *p* = 0.28, *η* ^2^ *p* = 0.006. Importantly, the interaction between age group and error type was significant, *F*(1,197) = 24.24, *p* < 0.001, *η* ^2^ *p* = 0.110, indicating that the proportion of isolated and consecutive errors were different between age groups. Post hoc paired‐sample *t*‐tests comparing error types for each age group revealed that children committed a higher proportion of consecutive errors compared to isolated errors, *t*(158) = 5.99, *p* < 0.001, *d* = 0.95 (Figure 3A). In contrast, adults committed fewer consecutive errors than isolated errors, *t*(39) = −13.85, *p* < 0.001, *d* = −4.43 (Figure 3B). The results of mixed‐design ANOVA on RTs with age group (children vs. adults) and error type (isolated vs. consecutive) did not reveal any significant effects of age, *F*(1,168) = 2.68, *p* = 0.10, *η* ^2^ *p* = 0.016, error type *F*(1,168) = 0.07, *p* = 0.78, *η* ^2^ *p* < 0.001 or their interaction *F*(1,168) = 0.09, *p* = 0.76, *η* ^2^ *p* < 0.001 (Figure 3C,D).

Lastly, we examined whether errors occurred more frequently during switch trials compared to repeat trials, and whether the distribution of isolated and consecutive errors differed in their proportions between the switch and repeat conditions. To this end, we further divided consecutive errors into three categories: the initial errors, the middle errors, and the last errors of each consecutive error series. This analysis was restricted to children, as adults exhibited a very low number of consecutive errors. A repeated‐measures ANOVA on percentage of errors among all responses, with the within‐subject factors error type (isolated, first, middle and last consecutive errors) and condition (switch vs. repeat) revealed a significant main effect of condition, *F*(1,158) = 124.25, *p* < 0.001, *η* ^2^ *p* = 0.440, indicating that errors in general occurred more frequently during switch trials (*M* = 5.13%, SD = 4.49) than repeat trials (*M* = 3.72%, SD = 3.69), consistent with the results above. The main effect of error type was also significant, *F*(3,474) = 68.51, *p* < 0.001, *η* ^2^ *p* = 0.302, as was the interaction between condition and error type, *F*(3,474) = 44.32, *p* < 0.001, *η* ^2^ *p* = 0.219. Post hoc paired‐samples *t*‐tests comparing conditions (switch vs. repeat) for each error type separately showed that the first errors of consecutive series occurred more frequently during switch trials (*M* = 5.17%, SD = 4.38) than during repeat trials (*M* = 3.49%, SD = 3.70), *t*(158) = 7.00, *p*_*Bonf*_. < 0.001, *d* = 1.11. Isolated errors also occurred more often during switch trials (*M* = 8.17%, SD = 3.23) than during repeat trials (*M* = 4.61%, SD = 2.47), *t*(158) = 11.55, *p*_*Bonf*_. < 0.001, *d* = 1.84. The difference between switch and repeat trials was not significant for the middle errors of consecutive error series (*M*_switch_ = 2.80%, SD_switch_ = 4.36, *M*_repeat_ = 2.75%, SD_repeat_ = 4.26), *t*(158) = 0.29, *p*_*Bonf*_. = 1, *d* = 0.05 nor for the last errors of consecutive error series (*M*_switch_ = 4.39%, SD_switch_ = 4.19, *M*_repeat_ = 4.04%, SD_repeat_ = 3.87), *t*(158) = 1.49, *p*_*Bonf*_. = 0.55, *d* = 0.24. Taken together, these results suggest that consecutive error series were more likely to start during switch trials than during repeat trials; however, the continuation and termination of these series were similar across conditions.

To gain a better understanding of whether isolated errors could be corrected due to the repetition of the relevant rule, we compared the percentage of correct trials following isolated errors between switch and repeat conditions. The result of the paired *t*‐test revealed that the occurrence of correct responses following isolated errors was more frequent during repeat trials (*M* = 6.67%, SD = 2.83) than during switch trials (*M* = 6.07%, SD = 2.63), *t*(158) = −2.21, *p* = 0.03, *d* = 0.35. These results suggest that post‐error adjustments were more effective when the task demand remained the same.

### 3.4 Age Differences in PES

**FIGURE 4:**
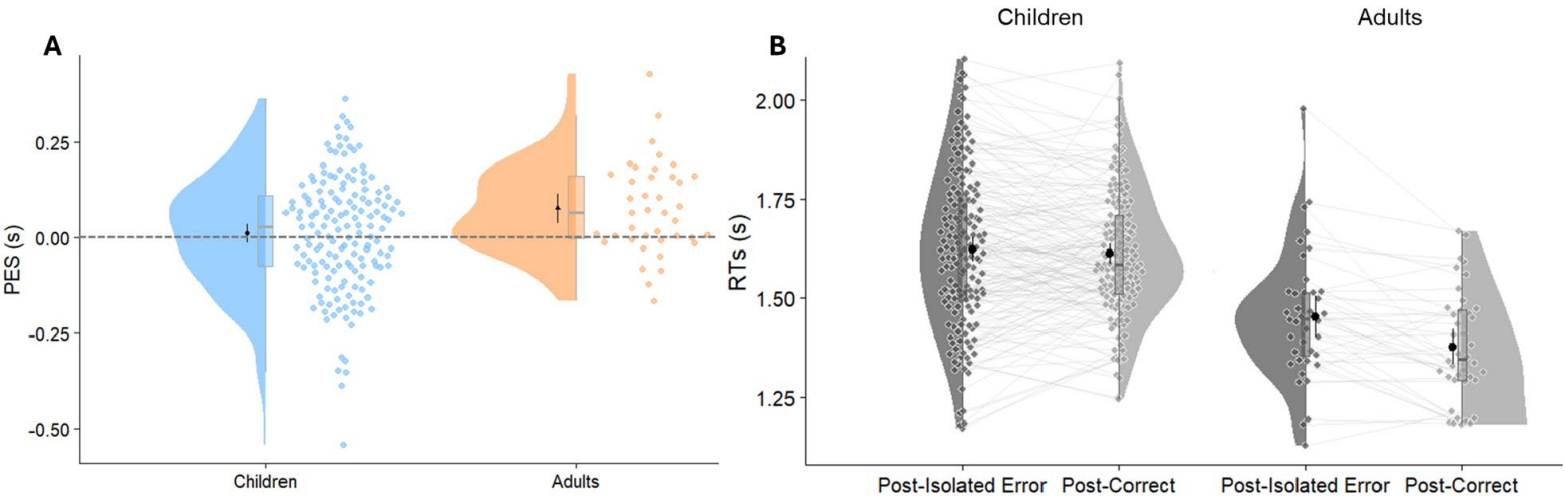
PES magnitude for children and adults. (A) PES between children and adults. (B) RT distributions for correct trials following isolated errors (post‐isolated error) and correct trials following correct trials (post‐correct) in children and adults. Each dot represents an individual participant connected by gray lines across conditions. Boxes show the interquartile range (IQR) with whiskers extending to 1.5 × IQR; horizontal lines within boxes indicate medians. Black dots represent means with 95% confidence intervals. Violin plots display response distributions.

Next, we investigated the degree to which PES differed between children and adults (see Figure 4A). The results of one‐sample *t*‐tests examining the presence of PES indicated that PES magnitude did not significantly differ from zero in children (*M* = 0.01, SD = 0.15), *t*(158) = 0.91, *p* = 0.36, *d* = 0.14. In contrast, in adults (*M* = 0.07, SD = 0.12) PES magnitude differed significantly from zero, *t*(39) = 4.02, *p* < 0.001, *d* = 1.29, indicating the presence of PES in this group. An independent sample *t*‐test comparing PES magnitudes between children and adults revealed a significant difference between age groups, *t*(73) = 2.91, *p* = 0.004, *d* = 0.68, confirming a more pronounced PES magnitude in adults (see Figure 4B).

PES might depend on the duration of interval between subsequent trials (i.e., ITI). Our experimental design with jittered ITIs allowed us to test whether variations in ITI affected PES. To this end, we conducted a control analysis to examine whether the magnitude of PES depends on variations in ITI in children and adults. A mixed‐design ANOVA on PES magnitude with age group (children vs. adults) and ITIs (1, 2, 4, and 6 s) revealed no significant main effect of age group, *F*(1, 48) = 2.90, *p* = 0.09, *η* ^2^ *p* = 0.057, ITIs, *F*(3, 144) = 1.75, *p* = 0.18, *η* ^2^ *p* = 0.035 or their interaction, *F*(3, 144) = 0.68, *p* = 0.52, *η* ^2^ *p* = 0.014. Together, these results indicate that varying ITIs in the present paradigm were unlikely to contribute to the observed age differences in PES magnitude.

Finally, to further examine the association between slowing and subsequent accuracy in children, we conducted a generalized linear mixed‐effects model at the trial level, with current accuracy predicted by the interaction between current RT and previous trial accuracy. The results revealed a significant interaction between response time and previous trial accuracy, *b* = −0.28, SE = 0.07, *p* < 0.001. Furthermore, we observed a main effect of previous trial accuracy on current accuracy, *b* = 0.66, SE = 0.11, *p* < 0.001, but no main effect of current response time on accuracy, *b* = 0.08, SE = 0.06, *p* = 0.17. To follow up on the interaction, we conducted post hoc generalized linear mixed‐effects models separately for previously correct and incorrect trials. The results revealed a significant negative effect of response time on accuracy when the previous response was correct, *b* = −0.22, SE = 0.04, *p* < 0.001, and a significant positive effect when the previous response was an error, *b* = 0.13, SE = 0.06, *p* = 0.03. Thus, when the previous trial was an error, response times were slower for correct current trials than incorrect current trials. In contrast, when the previous trial was correct, response times were faster for correct current trials than incorrect current trials.

### 3.5 Correlations Among PES, Accuracy and Switch Costs

**FIGURE 5:**
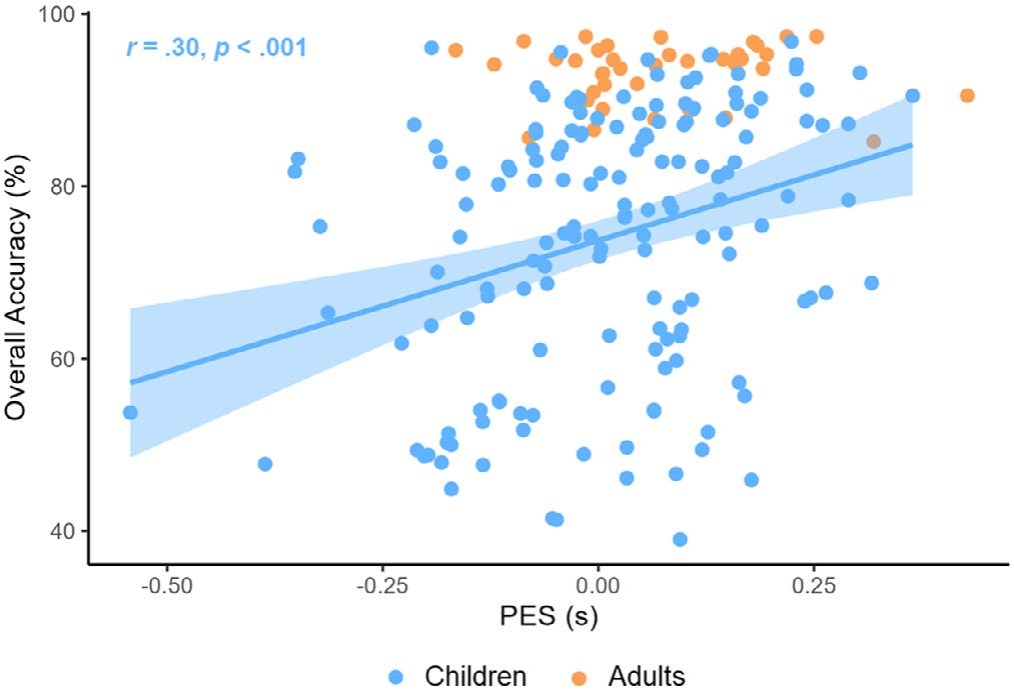
Correlation between PES and task‐switching performance in children and adults. Greater PES was associated with higher overall accuracy in children but not adults. Shaded areas represent 95% confidence intervals around the regression line for the children.

We then explored whether adults and children who exhibited greater PES also demonstrated higher accuracy, which would be indicative of an adaptive PES response. In children, a significant positive correlation was observed, *r*(157) = 0.30, *p* < 0.001, indicating that children who showed overall greater PES magnitudes showed higher accuracy during task‐switching. No significant correlation was found between PES magnitude and overall accuracy in adults, *r*(38) = −0.03, *p* = 0.87 (see Figure 5). A Fisher's *z*‐test comparing the correlations between children and adults revealed a reliable difference between these correlations (*p* = 0.01). However, the interpretation of the difference in correlations is limited by the high accuracy and limited variability in accuracy among adult participants.

Next, we investigated whether PES magnitude was specifically related to task switching. In children, we found no significant correlations between PES magnitude and switch costs in accuracy, *r*(157) = −0.13, *p*_*Bonf*_. = 0.17, nor between PES magnitude and RT switch costs, *r*(157) = 0.11, *p*_*Bonf*_. = 0.29. Similarly, in adults, there were no significant correlations between PES magnitude and switch costs in accuracy, *r*(38) = 0.11, *p*_*Bonf*_. = 0.97, or in RTs, *r*(38) = 0.07, *p*_*Bonf*_. = 1.

Taken together, these results suggest that greater PES was associated with better overall accuracy in children, potentially suggesting that PES may reflect a more general post‐error adjustment mechanism rather than one specifically tied to task‐switching performance.

In summary, age‐related differences emerged in different aspects of behavioral task performance. Adults outperformed children in overall accuracy and response times, while both groups showed slower responses for errors than correct trials. Children exhibited larger switch costs in accuracy and were more prone to consecutive error sequences than isolated errors. Moreover, PES was found in adults but absent in children; however, PES in children was associated with higher overall accuracy, suggesting adaptive post‐error adjustments.

### 3.6 fMRI Results

**FIGURE 6:**
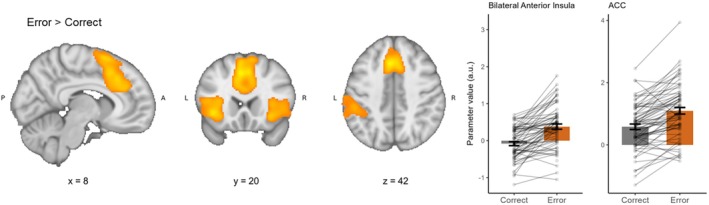
Results of whole‐brain analysis of error‐related activation in children. Whole‐brain analysis revealed significantly greater activation for error trials compared to correct trials in the ACC, bilateral anterior insula/frontal operculum (FO), and SMG (*p* < 0.001, cluster‐level FDR corrected (*p* < 0.05)). Only relevant regions which spheres around the peak activation were included in the ROI are shown. Bar plots visualize parameter estimates for correct and error trials in the bilateral anterior insula and ACC. Individual participant values are indicated with gray lines, and error bars represent the standard error of the mean.

To examine the neural correlates of error processing in children, we conducted a whole‐brain analysis of error‐related activity (error > correct, *p* < 0.001, cluster‐level FDR corrected (*p* < 0.05)). The results revealed significant activation in the bilateral dorsal ACC, bilateral anterior insula/frontal operculum, and left supramarginal gyrus (SMG) (for peak activations see Table 1). These results highlight the robust engagement of error‐monitoring regions, including the bilateral dACC and bilateral anterior insula/frontal operculum during error processing in children (Figure 6). The contrast of correct > error trials elicited greater activation in the right and left putamen, left superior temporal gyrus, left inferior occipital gyrus, and occipital pole (see Table 1).

**FIGURE 7:**
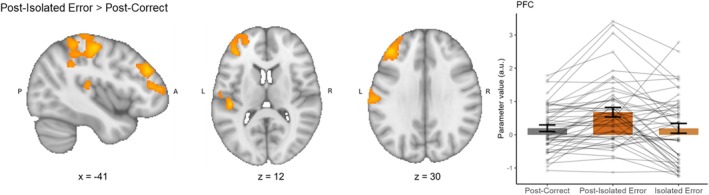
Results of whole‐brain analysis of post‐isolated error‐related activation in children. Whole‐brain analysis results revealed significantly greater activation for correct responses following isolated errors (post‐isolated error) compared to correct responses following correct responses (post‐correct) in the PFC, cerebellum, and postcentral gyrus. Only relevant regions which spheres around the peak activation were included in the ROI are shown (*p* < 0.001, cluster‐level FDR corrected (*p* < 0.05)). Bar plots on the right visualize the effect and show parameter estimates for post‐correct, post‐isolated error, and isolated‐error trials in the lateral PFC. Individual participant values are indicated with gray lines, and error bars represent the standard error of the mean.

To examine the neural correlates of post‐error processing in children, we conducted a whole‐brain analysis comparing correct responses following isolated errors (post‐isolated error) and correct responses following correct responses (post‐correct). For the contrast post‐isolated error > post‐correct (voxel‐wise threshold *p* < 0.001, cluster‐level FDR correction *p* < 0.05), the results revealed significant activation in the postcentral gyrus, right cerebellum, and lateral PFC (for peak activations see Table 2). These results highlight the engagement of PFC during post‐error processing in children (Figure 7). The contrast of post‐correct > post‐isolated error trials elicited greater activation in the left inferior occipital gyrus (see Table 2).

### 3.7 Brain‐Behavior Correlation Analysis

**FIGURE 8:**
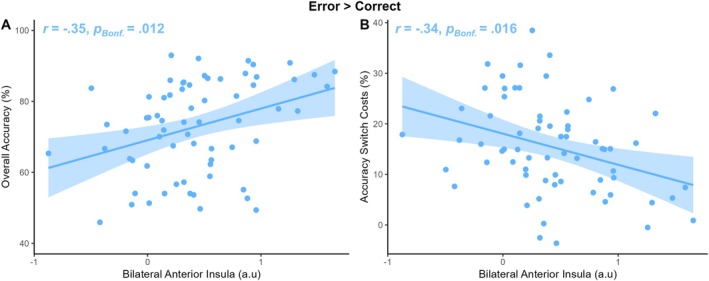
Associations between error‐related anterior insula activity and behavioral measures. (A) A significant positive correlation was observed between error‐related bilateral anterior insula activity and overall task accuracy indicating that greater insula engagement was associated with higher accuracy. (B) A significant negative correlation was observed between error‐related bilateral anterior insula activity and switch costs in accuracy indicating that greater insula engagement was associated with lower switch costs in accuracy. Shaded areas represent 95% confidence intervals around the regression lines.

We next examined the association between behavioral performance measures including PES, overall task performance and accuracy switch costs with error‐related brain activation (ACC and bilateral anterior insula for the contrast error > correct) and post error‐related brain activation (PFC for the contrast post‐isolated error > post‐correct). There were positive associations between bilateral anterior insula activation and both overall accuracy, *r*(65) = 0.35, *p*_*Bonf*_. = 0.012 (see Figure 8A) and PES magnitude (see Figure 8B) at trend level, *r*(65) = 0.22, *p*_*Bonf*_. = 0.28. Critically, we observed a negative correlation between error‐related activity in the anterior insula and accuracy switch costs, *r*(65) = −0.34, *p*_*Bonf*_. = 0.016. There were no associations with RT switch costs, *r*(65) = 0.05, *p*_*Bonf*_. = 1. No significant correlations were observed between brain activation in ACC and PFC, and any behavioral measures (all *p* values > 0.20). Together, these results suggest that error processing in the bilateral anterior insula contributed to adaptive performance adjustments during task switching.

## 4 Discussion

This study investigated age differences in PES and different error types between children and adults. We further linked individual differences in PES to differences in the neural correlates of error processing during task switching. Our results revealed developmental differences in error correction behavior between adults and children in the context of task switching. Whereas adults were more likely to correct errors immediately, resulting in predominantly isolated errors, children were more likely to make consecutive errors, indicating potentially ongoing development in behavioral adjustments following an error. The higher occurrence of consecutive errors compared to isolated errors among children suggests difficulty implementing effective error correction and making quick adjustments in behavior within the context of the present task‐switching paradigm. These findings align with previous results demonstrating that children are less efficient than adults in monitoring their errors (Davies et al. 2004; Wiersema et al. 2007) and adjusting their behavior accordingly (Best and Miller 2010; Davidson et al. 2006; Diamond 2013; Zelazo et al. 2008).

Our results also revealed that both age groups executed error responses more slowly than correct responses. This difference is expected in the present paradigm: in children and adults, errors are typically faster than correct responses in tasks requiring speeded responses (Davies et al. 2004; Wiersema et al. 2007), where errors result from impulsive or premature responses before full evaluation of information (Gehring et al. 1993; Hester et al. 2004; Rabbitt 1966). However, in more complex tasks such as the present one, errors are often slower than correct responses, because they are more likely to result from uncertainty and weak or ambiguous representations of relevant information (Maddox et al. 1998; Ratcliff and Rouder 1998). Critically, the effect of slower error responses in comparison to correct ones was more pronounced in adults, possibly reflecting adults' increased sensitivity to response uncertainty relative to children.

Beyond these differences in errors, our results also demonstrated lower or even absent post‐error slowing in children relative to adults, suggesting that children may not engage in the strategic behavioral adjustment that PES is thought to reflect to the same extent as adults (Carrasco et al. 2013; Dubravac et al. 2022; Fairweather 1978; Gupta et al. 2009). Finally, among children, those with more pronounced PES magnitude demonstrated higher overall task accuracy, suggesting that PES plays an adaptive role in task performance. Supporting this, additional analyses demonstrated that PES is meaningfully associated with improved subsequent accuracy, supporting the interpretation of PES as an adaptive process in the present task‐switching.

The present findings diverge from the findings of most previous studies on PES in childhood (e.g., Gupta et al. 2009; Schachar et al. 2004), which have shown decreasing PES with increasing age. Rather, our results corroborate studies that found lower PES in children in comparison to adults (Friedman et al. 2009; Hogan et al. 2005). Parts of the inconsistencies in developmental findings might stem from variations in study designs in which PES has been measured. One such variation concerns ITIs: while some experiments used ITIs of fixed duration, the duration of these ITIs varied across studies, ranging from as short as 100 ms to as long as 1400 ms (e.g., Dubravac et al. 2022; Ger and Roebers 2023). Other studies employed jittered ITIs of different ranges (Friedman et al. 2009; Overbye et al. 2019; Wiersema et al. 2007) and, similarly to us, found higher PES with older age. ITIs are important when considering age differences in PES, as children may need more time than adults between error and post‐error trials to engage in effective strategic adjustment (Wessel 2018). If so, one might expect that PES will increase with longer ITIs. However, our control analyses didn't reveal any ITI effects on PES in children or adults. Those exploratory analyses should be treated with caution as longer ITIs were less frequent than shorter ones, which may have induced differences in the expectation and strategies adopted by the participants. Future research is needed to systematically examine PES across various combinations of ITIs, stimulus durations, and types of tasks.

Additionally, the duration of stimulus presentation varies considerably across previous studies, from very short intervals (e.g., 100 ms) to considerably longer ones (e.g., self‐paced). Such differences may lead to different age effects because shorter stimulus durations, similar to shorter ITIs, may limit the time available for error detection and adjustment, which would particularly affect children. In contrast, longer stimulus presentation may provide more opportunity for post‐error adjustments, potentially amplifying PES effects in children compared to adults, since children may require more time to implement such adjustments. As mentioned earlier, studies reporting decreases in PES with age tend to use longer stimulus durations (de Mooij et al. 2022; Dubravac et al. 2022; Ger and Roebers 2023; Gupta et al. 2009; Jones et al. 2003; Schachar et al. 2004), while those reporting increases in PES with age often employ shorter stimulus durations (Davies et al. 2004; Friedman et al. 2009; Hogan et al. 2005; Wiersema et al. 2007). Contrary to expectations, we found a pattern more consistent with studies using shorter stimulus durations that reported increases in PES with age, even though our stimulus presentation time was comparable to studies that reported decreases in PES with age. However, due to the complexity of our three‐rule task‐switching paradigm, it is possible that our 2000 ms presentation time did not functionally act as a “long” duration in the same way as truly self‐paced or less demanding tasks (e.g., Dubravac et al. 2022; Gupta et al. 2009; Jones et al. 2003), thereby allowing us to capture the still‐developing PES in children. Future research is needed to test timing needed for strategic adjustments in different age groups of children in relation to different task demands.

In the present task‐switching paradigm, the absence of PES in children may reflect the interplay of several developing processes, including cognitive control adjustments (Dubravac et al. 2022; Gupta et al. 2009), metacognitive awareness (Destan et al. 2014; Steiner et al. 2020), and/or working memory capacity (Gathercole et al. 2004; Reynolds et al. 2022). These developmental differences may constrain children's capacity to maintain task‐relevant information and rules, to detect their errors and accurately evaluate their actions, and to flexibly adjust their behavior. Together, they may limit the cognitive resources available for adaptive regulation and thus lead to less effective post‐error adjustments. According to Wessel (2018), adaptation processes follow an automatic cascade, where errors trigger ongoing cognitive processes that facilitate the identification of the source of the expectation violation. In classical response time experiments, identifying the source of the violation may happen quickly, as there are limited possibilities, such as misremembering the stimulus–response mapping or experiencing an attention slip. In the present study, due to the relative complexity of the task, there were several additional possible sources of expectation violations: the cue–task mappings and target stimulus–response mappings could be remembered incorrectly, especially as there were three options for cues, stimuli, and responses; the location of the target stimulus varied randomly across trials such that detecting the target stimulus may have posed additional difficulty. Taken together, the adaptation processes in the present task‐switching paradigm were relatively more demanding, potentially leading to less possibility to express effective PES.

Finally, PES seems to be influenced by the conscious perception of an error (Wessel 2018) such that action errors that are not detected as such do not result in PES. The absence of feedback in our design may have hampered children's error detection and initiation of corrective actions. Consistent with this interpretation, children in our study executed more consecutive errors than isolated errors, which might suggest that they had difficulty interrupting an ongoing sequence of errors due to delayed detection of their errors. This aligns with established findings that metacognitive skills continue to develop across childhood and adolescence (Fandakova et al. 2017; Veenman et al. 2006). Similarly, the development of meta‐control, the ability to monitor and regulate the cognitive control strategies (Eppinger et al. 2021), also improves across childhood and adolescence (Bolenz and Eppinger 2022; Chevalier 2015).

Among children in the present study significant activation for errors compared to correct responses was observed in brain regions typically associated with error processing such as the ACC (Checa et al. 2014; Tamnes et al. 2013; Van Veen and Carter 2006) and the anterior insula (Fitzgerald et al. 2010; Rubia et al. 2006; Uddin et al. 2011). The cingulo‐opercular network, encompassing the ACC, insula, anterior PFC, and thalamus, plays a key role in maintaining a stable response state, which helps to consistently and flexibly apply behavioral adjustments to meet task demands (Luna et al. 2010). Within this network, error‐related activation in the anterior insula was positively associated with higher overall accuracy and lower switch costs, potentially reflecting the key role of error processing for flexible task switching among children. Our findings regarding anterior insula involvement in error processing and post‐error adjustments are largely consistent with previous research. The role of the anterior insula during PES (Zhang et al. 2017) and behavioral adjustments is well established (see also Kerns et al. 2004; Cavanagh et al. 2009). Ullsperger et al. (2010) further discussed that anterior insula activation during performance monitoring is modulated by error awareness. In contrast, the ACC appears to be active during both aware and unaware errors (Hester et al. 2007). In line with the discussion above, enhanced error‐related activity in the present study may be linked to more efficient error detection, allowing for the cascade of strategic adjustments to be initiated. Future studies examining the role of feedback on error processing, perception among children can help further understand the factors contributing to the development of the ability to adjust behavior and learn from errors.

Extending this focus to post‐error processing, we observed increased lateral PFC activation during correct trials following error trials in comparison to correct trials following correct trials. The cluster we observed encompassed the anterior and middle frontal gyrus, corresponding to dorsolateral and anterior PFC. Post‐error engagement of these PFC regions has been consistently reported in previous fMRI studies and is thought to reflect behavioral adjustment and the engagement of cognitive control (Garavan 2002; Hester et al. 2007; King et al. 2010; Li et al. 2008; Zhang et al. 2017). Enhanced PFC activation following an error has also been directly linked to PES (Cavanagh et al. 2009; Debener et al. 2005). For example, Garavan (2002) demonstrated that activation in dorsolateral PFC emerged specifically when response times slowed down after errors and was absent when responses remained fast. Moreover, PFC activation was greater in individuals who exhibited a larger adjustment in their response speed, indicating a functional relationship between PFC engagement and post‐error control (Garavan 2002). Developmental evidence further supports this interpretation. Fandakova et al. (2018) reported increased anterior PFC activation during uncertain compared to incorrect trials in older children (10–12 years) and adults and showed that anterior PFC involvement in uncertainty‐related processing is not fully mature until middle childhood. Our results align with these findings and support the interpretation that errors in the present task are more likely to reflect uncertainty arising from incomplete or unstable memory representations of the task rules, which subsequently engages prefrontal control mechanisms in the service of monitoring processes (Burgess and Wu 2012; Fleming and Dolan 2012; Koechlin and Hyafil 2007) to guide post‐error adjustments.

In sum, our findings suggest that, in the context of task switching, children's performance is characterized by developing cognitive control dynamics, reflected in the absence of PES and an increased tendency toward consecutive errors, rather than immediate post‐error adjustments as observed in adults. This age difference pattern may indicate difficulties in effectively processing and monitoring errors. Accordingly, at the neural level, the association between anterior insula activity, overall accuracy, and switch costs points to its role in supporting error monitoring, potentially through error detection. Together, these results highlight that the mechanisms linking neural error signals to behavioral adjustments continue to mature throughout late childhood.

## Author Contributions

**Gülce Akin:** data curation, formal analysis, methodology, visualization, writing – original draft, writing – review and editing. **Sina A. Schwarze:** data curation, investigation, methodology, writing – review and editing. **Ulman Lindenberger:** conceptualization, methodology, resources, writing – review and editing. **Silvia A. Bunge:** conceptualization, methodology, writing – review and editing. **Yana Fandakova:** conceptualization, data curation, funding acquisition, investigation, methodology, project administration, resources, supervision, writing – review and editing.

## Funding

This work was supported by Deutsche Forschungsgemeinschaft, FA 1196/2‐1 and by the Jacobs Foundation, 2023‐1510‐00.

## Data Availability

The data supporting the results of this study can be obtained from the corresponding authors upon reasonable request.
